# Complete, Rapid Resolution of Severe Bilateral Pneumonia and Acute Respiratory Distress Syndrome in a COVID-19 Patient: Role for a Unique Therapeutic Combination of Inhalations With Bromhexine, Higher Doses of Colchicine, and Hymecromone

**DOI:** 10.7759/cureus.30269

**Published:** 2022-10-13

**Authors:** Tsanko Mondeshki, Radoslav Bilyukov, Toma Tomov, Miroslav Mihaylov, Vanyo Mitev

**Affiliations:** 1 Department of Propaedeutics of Internal Diseases, University Hospital Alexandrovska, Medical University-Sofia, Sofia, BGR; 2 COVID-19 Unit, University Hospital Alexandrovska, Sofia, BGR; 3 Department of Pulmonary Disease, University Hospital Alexandrovska, Medical University-Sofia, Sofia, BGR; 4 Department of Chemistry and Biochemistry, Medical University-Sofia, Sofia, BGR

**Keywords:** hyaluronan storm, bradykinin storm, cytokine storm, nlrp3 inflammasome, hymecromone, colchicine, bromhexine, covid-19

## Abstract

An otherwise healthy, 35-year-old man was hospitalized for the management of acute respiratory failure due to coronavirus disease 2019 (COVID-19)-related severe bilateral pneumonia and acute respiratory distress syndrome (ARDS). The patient therapeutic regimen included the widely accepted standard combination of oxygen, anticoagulation therapy; corticosteroids, non-steroidal anti-inflammatory drugs (NSAIDs), and antibiotics. A novel combination of colchicine, hymecromone, and bromhexine inhalations was added to the therapeutic regimen as part of our unique COVID-19 management institutional protocol. COVID-19-related severe bilateral pneumonia and acute respiratory distress syndrome (ARDS). The patient therapeutic regimen included the widely accepted standard combination of oxygen, anticoagulation therapy, corticosteroids, NSAIDs, and antibiotics. A novel combination of colchicine, hymecromone, and bromhexine inhalations was added to the therapeutic regimen as part of our unique COVID-19 management institutional protocol.

Rapid clinical response on day 2, with a significant improvement of radiographic pulmonary changes on day 5, and improvement of laboratory results on days 5-7 were observed. The administration of inhalatory bromhexine in combination with high-dose colchicine and hymecromone was crucial for the positive outcome of the disease. This treatment regimen resulted in a four to five-fold decrease in the mortality of hospitalized patients.

## Introduction

The coronavirus disease 2019 (COVID-19) pandemic has been raging for nearly three years, and despite tremendous advances in vaccination strategies worldwide, the question of effective treatment options remains widely unanswered.

The biochemical mechanisms of severe acute respiratory syndrome coronavirus-2 (SARS-CoV-2) action have been studied in detail. It has been shown that the virus gains access to the cell by the action of several proteases (furin, TMPRSS2 (transmembrane protease serine subtype 2), etc.) [1]. One important implication of these findings is that some protease inhibitors could play a critical role in the treatment of the disease. One such protease inhibitor that can be used in the treatment regime is the well-known, affordable drug bromhexine hydrochloride, which has minimal side effects [1].

It has been shown that the virus can stimulate the formation of NLRP3 (Nod-like receptor family, pyrin domain containing 3) inflammasome, which, in turn, activates caspase-1. Activated caspase-1 leads to the conversion of pro-interleukin IL-1β and pro-IL-18 into their active forms. Finally, IL-1β stimulates the synthesis of IL-6, a major proinflammatory factor in the cytokine storm related to COVID-19 [2]. Recent studies have shown that the anti-inflammatory agent colchicine can alleviate the cytokine storm by inhibiting the inflammasome and IL-1β [3].

Another very specific feature of SARS-CoV-2 viral infection is the accumulation of hyaluronic acid (HA) in the lung alveoli, leading to hyaluronan storm syndrome and eventually to acute respiratory distress syndrome (ARDS) in high-risk COVID-19 patients [4]. Several studies have recently shown that coumarin derivative, 4-Methylumbelliferone (4-MU; hymecromone) can decrease the accumulation of HA and thus prevent the hyaluronan storm by inhibiting the first step of HA synthesis [3,4].

ARDS is characterized by massive alveolar damage, pulmonary edema, hyaline membrane formation, and progressive respiratory failure. These pathological features are characteristic of SARS and Middle Eastern respiratory syndrome (MERS) coronavirus infection [4].

We demonstrate in this case that the unique combination therapy with inhalatory bromhexine hydrochloride, high-dose colchicine, and hymecromone added to the standard COVID-19 treatment regimens resulted in rapid clinical and paraclinical improvement and the resolution of a complicated case of COVID-19 pneumonia and severe ARDS. Our large-scale clinical data showed that the utilization of this treatment regimen led to a five-fold decrease in inpatient mortality rate.

## Case presentation

A 35-year-old, otherwise healthy man was seen by his primary care physician for worsening sore throat, muscle stiffness, and fever for two days. He tested positive for SARS-CoV-2 on a nasal swab polymerase chain reaction (PCR) test and was prescribed azithromycin (500 mg/day), nonsteroidal anti-inflammatory drug nimesulide (100 mg, two times a day, and colchicine (0.5 mg, three times a day). He felt initially better and thus decided to stop nimesulide and reduced the dose of colchicine to 0.5 mg daily. However, after a short initial improvement, his overall condition quickly worsened and, on day 7, he presented to the emergency department with severe headaches, high-grade fevers, shortness of breath on exertion, and oxygen saturation as low as 74%.

He was admitted to the hospital with acute respiratory failure for further management and monitoring. His past medical and family histories were noncontributory, he was not taking any prescribed medications, and he did not report any allergies to food and medication. He is a never-smoker and drank alcohol on social occasions. He denied using any medications chronically. He worked as a software specialist.

**Figure 1:**
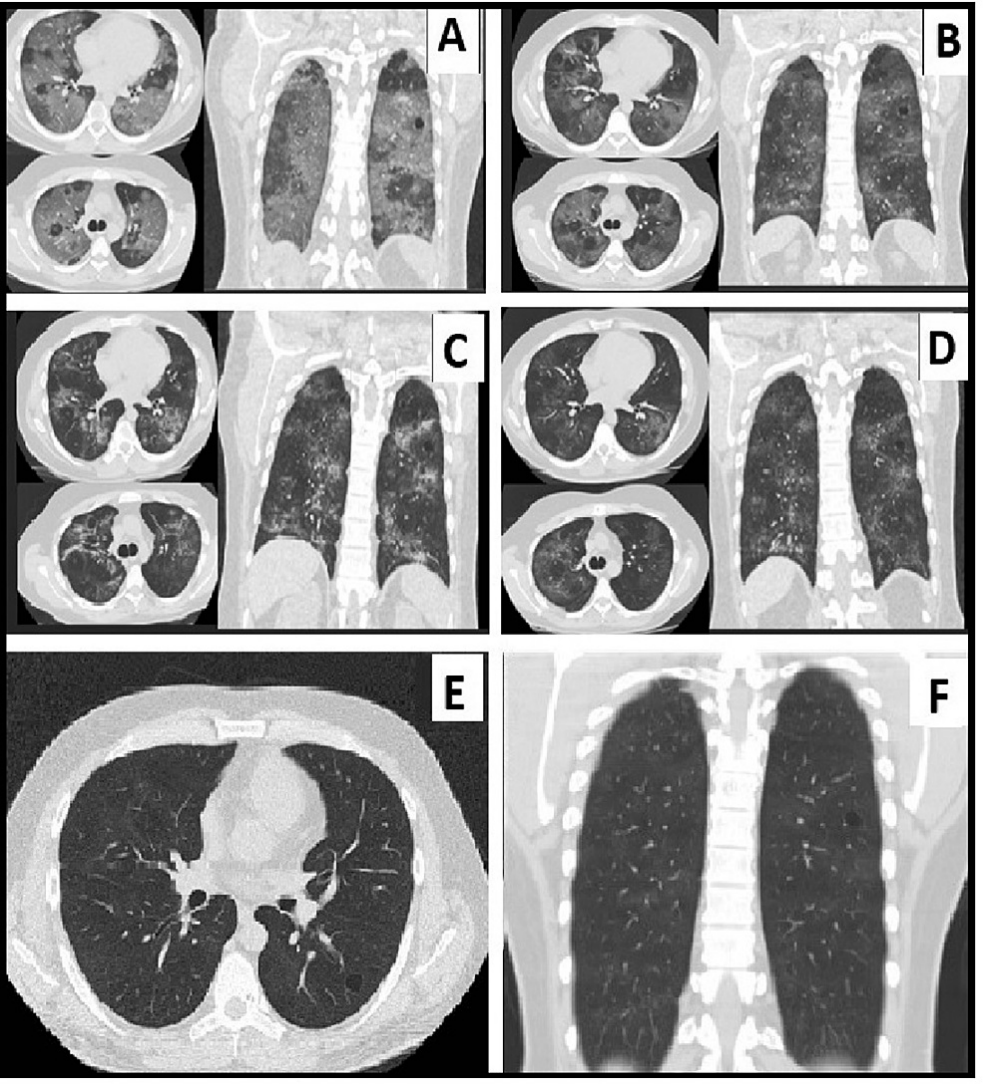
Dynamics of the radiological changes on chest CT A - axial and coronal views with widespread ground-glass opacities at presentation, B - axial and coronal views on day 5 after the initiation of therapy, C - axial and coronal views on day 11 after the treatment, D - axial and coronal views on patient’s discharge (day 27), E - axial view and F - coronal view - outpatient follow-up images showing a complete resolution of infiltrative changes

The workup on admission, including extensive laboratory studies and chest computed tomography, revealed severe bilateral lung infiltrative changes (ground-glass opacities type) highly suspicious for SARS-CoV-2 pneumonia (Figure 1A). Arterial blood gas analysis demonstrated respiratory alkalosis with significant hypoxia with hypocapnia. His laboratory blood workup was significant for leukocytosis (13.8x10^9/l), thrombocytosis (484x10^9/l), elevated D-dimers (0.97 mg/l), and inflammatory acute phase protein markers CRP (77 mg/l) and ferritin (1993 mkg/l) (Table 1).

**Table 1:** Laboratory test results that changed during the course of the disease

Examination	Range	Day 1	Day 3	Day 4	Day 5	Day 7	Day 10	Day 13	Day 15	Day 21	6 month
3350 - Procalcitonin 1 (ng/ml)		0.052	0.071				0.033		0.047	0.060	
4751 - COVID-19 (SARS-CoV-2 ribonucleic acid) PCR test		positive /+/									
Leukocytes (Leu) (10^9/l)	3.5-10.5	13.8	11.4	16.4	14.6	16.5	12.2	10.0	10.2	10.3	8.2
Haemoglobin (Hb) (g/l)	135-180	138	142	137	145	139	141	137	143	131	155
Hematocrit (Ht)	0.40-0.53	0.43	0.44	0.43	0.44	0.43	0.44	0.42	0.43	0.39	0.48
Red blood cell distribution width (RDW) (%)	11.5-14.5	13.7	13.6	13.4	13.3	13.3	13.1	13.1	12.9	13.5	14.1
Thrombocytes (Tr) (10^9/l)	130-440	484	469	468	471	308	266	105	249	167	386
Mean platelet volume (MPV) (fl)	6.1-11.5	9.5	9.2	9.4	9.1	10.1	10.7	12.1	9.9	9.1	9.1
NE % - Neutrophilic granulocytes %	40-70	87	79		82	88	89	85	76	84	58
LY % - Lymphocytes %	20-48	6	12		10	5	6	10	15	10	31
NE# - Neutrophilic granulocytes - number	2-7	12.0	9.0		11.9	14.6	10.9	8.4	7.7	8.7	4.8
MO# - Monocytes - number	0-0.8	0.8	0.8		1.2	1.0	0.5	0.5	0.9	0.5	0.5
LY# - Lymphocytes - number	1-4	0.9	1.3		1.4	0.9	0.8	1.0	1.6	1.0	2.5
IG (%) - Immature granulocytes %	0-5	9.3	11.8		5.3	1.6	0.9	1.1	1.4	1.4	0.4
Erythrocyte sedimentation rate* *(ESR) (mm/h)	0-18		20	12	18	12	3	3	2	3	
IG # - Immature granulocytes - number	0-0.7	1.3	1.3		0.8	0.3	0.1	0.1	0.1	0.1	0.0
Protein С/PrC/ %	54-166	113			157	136	144	179	200	196	
Protein S/PrS/ %	74-120	94			102	109	109	125	132		
Fibrinogen F-I (g/l)	2-4	4,2	4.9		3.7	3.0	2.8	2.9	2.2	2.6	
D-dimer (mg/l)	0-0.55	0.97	1.3	0.67	0.47	0.88	0.34	0.41	0.27	0.27	0.27
Ph	7.35-7.45	7.45		7.47	7.46						
pCO_2 _(kPa)	4.67-6	4.45		4.55	4.81						
pO_2 _(kPa)	10-13	6.71		19.24	24.75						
Standard serum bicarbonate (SB) (mmol/l)	21-25	24.9		25.90	26.2						
O_2_ sat (%)	94-98	81		99	99	98	97	97	95	96	
Aspartate aminotransferase (ASAT) - serum (U/l)	0-40	62		48	81	59	20	20	27	38	
Total protein - serum (g/l)	64-83	70	63	68	64	63	62	61	65	60	
Sodium (mmol/l)	136-151	145	141	139	137		138	132	133	132	141
Alanine aminotransferase (ALAT) - serum (U/l)	0-41	327	232	198	219	252	143	100	92	127	21
Glucose - serum (mmol/l)	3.6-6.1	9.2			8.7		9.6		6.2	9.2	5.5
Creatine phosphokinase - serum (U/l)	0-190	154	126		205		50	32	29	29	
Lactate dehydrogenase(LDH) - serum (U/l)	240-480	1072	1099	1009	801	598	452	465	384	399	259
Creatine phosphokinase - MB - serum (U/l)	0-25	24	26		22		15	19	14	14	10
Creatinine - serum (mkmol/l)	62-106	72	67	65	54		56	60	55	64	81
Gamma-glutamyl transferase (GGT) - serum (U/l)	0-60	172			183		142	115	101	101	
NT pro-BNP (pg/ml)	0-320	1490	1375			91.7	54.6	67.8	105	72.7	
CRP (mg/l)	5	77	27.1		6.7	3.1	1.8	0.9	0.7	3.8	6.9
Ferritin (mkg/l)	400	1993	1488	1501	1190	1349	1333	1356	1235	977.8	142

Our decision to include the combination of hymecromone, bromhexine, and colchicine in the standard therapeutic regimen was based on our current knowledge of viral biology and the biochemical mechanism of SARS-CoV-2.

Our unique combination therapy added to the standard therapeutic regimen included:

- Hymecromone, 2400 mg, (p.o.) daily,

- Bromhexine, nebulizers 8mg/TID,

- Ambroxol 30mg p.o./daily,

- Colchicine, initial loading dose of 0.5 mg/hour for the first five hours followed by 0.5 mg p.o. every 4 hours (maximum daily dose of 4.5 mg)

Standard treatment regimen:

- Oxygen therapy delivered by non-rebreather mask, 16 L/min

- Ketoprofen, 100 mg), intravenously (IV), daily

- Heparin drip (activated partial thromboplastin time target of 45-65 sec),

-Methylprednisolone, 125 mg IV every six hours on day 1, tapered down to 60 mg i.v. every six hours over the next four days with the addition of dexamethasone 4 mg i.v. every 12 hours,

- Vit D3,5000E, daily (p.o.),

- Meronem 3 x 1.0 g, IV,

- Continuous i.v. infusion of 0.9% normal saline

On day two of hospitalization, the general condition of the patient improved with a single low-grade fever of 37.5 ^o^C and oxygen saturation in the lower 90% on 12-15 L/min oxygen via a non-rebreather mask. On day three, the patient reported diarrhea, and the colchicine dose was reduced to 0.5 mg every six hours.

The patient’s general condition continued improving and his acute respiratory insufficiency, inflammatory markers, arterial blood gas analysis, and radiological images have nearly normalized on days 5-7 (Figures 1B, 1C). He was eventually discharged to home on day 27 and prescribed continue outpatient therapy with colchicine 0.5 mg, p.o., TID for 40 days, hymecromone 400 mg (p.o.) for 20 days, apixaban 5 mg, (p.o.) BID for 60 days, Vit. D3 2000E daily for 60 days, and itraconazole 100 mg (p.o.) daily for 15 days. The patient was discharged on day 27 with a nearly complete resolution of bilateral pneumonia (Figure 1D). The patient had no residual cough or any other symptoms on the follow-up visits two weeks after he was discharged. The six-month outpatient follow-up labs (Table 1) and CT of the chest (Figure 1E, 1F) revealed a complete resolution of the lung infiltrates.

## Discussion

Despite tremendous advances in vaccination strategies worldwide, the question of effective treatment options for COVID-19 remains largely unsolved [2-4].

**Figure 2:**
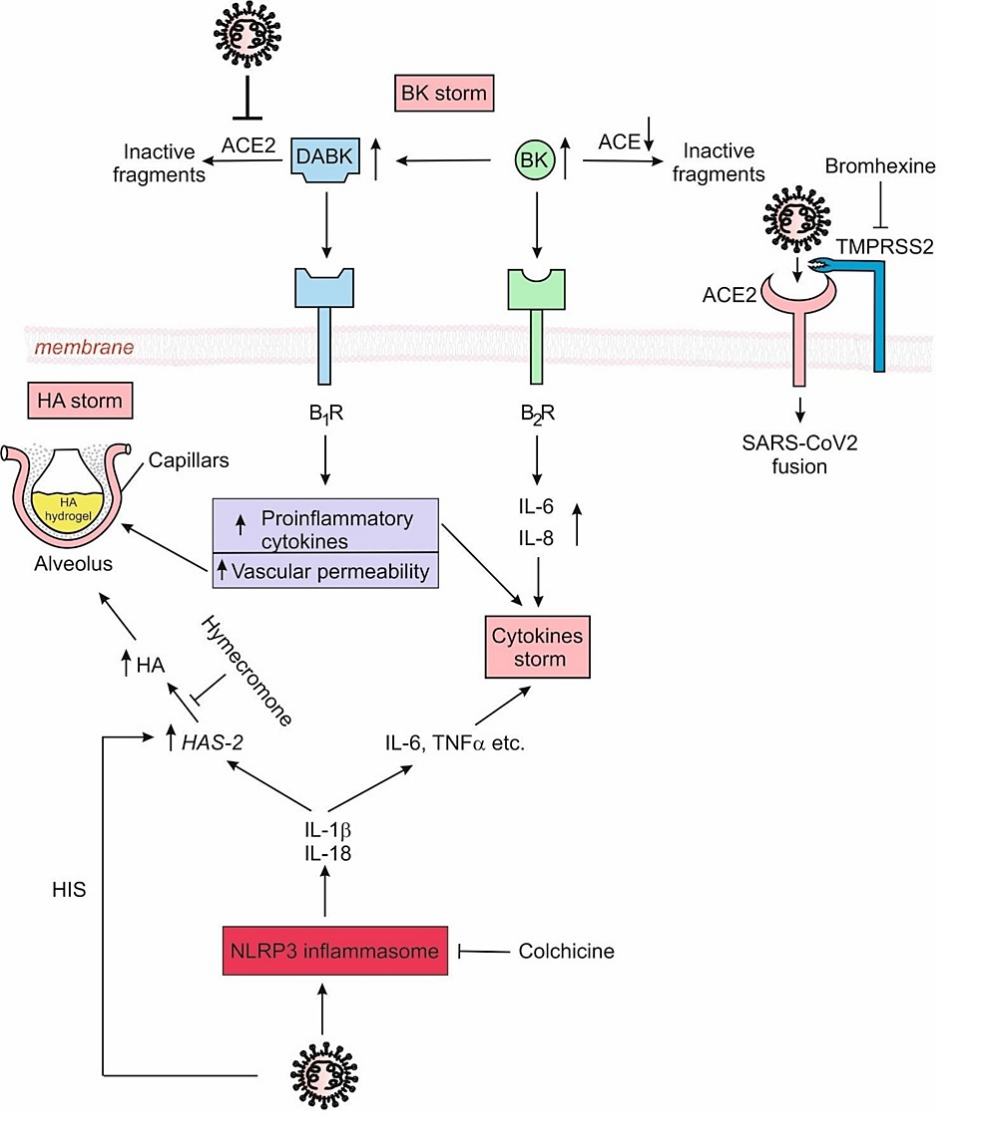
Vicious circle in COVID-19 complications Figure 2 is our creation and represents the biochemical pathogenesis of COVID-19. SARS-CoV-2 induces the NLRP3 inflammasome and that could result in its hyperactivation, followed by the overexpression of cytokines (CS). The high cytokine levels are strong inducers of HAS2 leading to a dramatic increase in HA. LMW-HA and oligo-HA could further enhance the CS effect by stimulating the cytokine release from immune and pulmonary cells. Following the binding of SARS-CoV-2 to ACE2, the levels of BR and DABK increase (BS), resulting in vasodilation and leakage of HA into the alveoli (HA storm). The BR storm is also associated with the upregulation of proinflammatory cytokines, strengthening the CS. These feedback loops are a typical vicious circle. The interruption of the vicious circle, which is the reason for СOVID-19 complications is the theoretical basis of our therapeutic approach. COVID-19: coronavirus disease 2019; SARS-CoV-2: severe acute respiratory syndrome coronavirus-2; HA: hyaluronic acid; CS: cytokine storm; BR: bradykinin; DABK: des-Arg (9)-bradykinin

The logic behind our therapeutic method is based on current knowledge of the biochemical mechanism of SARS-CoV-2 (Figure 2).

It has been convincingly shown that the entry of SARS-CoV-2 into the cells is mediated by the binding of the viral spike (S) glycoprotein to the angiotensin-converting enzyme 2 (ACE2) receptor in the cells, followed by the proteolytic cleavage of the S glycoprotein by the host proteases, including furin and TMPRSS2 [1]. Thus, it is logical that inhibiting these proteases will limit the cellular internalization of the virus and, as a result, will reduce the clinical severity. It has been shown that bromhexine hydrochloride (BRH) (an over-the-counter, safe, and inexpensive drug) acts as a mucolytic and is a selective inhibitor of TMPRSS2 [5]. The lack of a distinct effect of BRH tablets is due to the route of administration [6]. Valuable time is wasted until BRH saturation is achieved, during which time the virus penetrates and multiplies.

One of the main pathological events of SARS-CoV-2 is the induction and hyperactivation of the NLRP3 inflammasome, which leads to increased levels of pro-inflammatory cytokines (cytokine storm - CS). The high cytokine levels induce hyaluronan synthase 2 (HAS2) leading to a dramatic increase in HA. Low molecular weight HA (LMW-HA) and oligo-HA could further enhance the CS effect by stimulating the cytokine release from pulmocytes and immune cells. Following the binding of SARS-CoV-2 to ACE 2, the levels of bradykinin (BR) and des-Arg (9)-bradykinin (DABK) increase significantly, which is also known as bradykinin storm (BS). BS leads to vasodilation and HA accumulation in the alveoli (HA storm) and the upregulation of proinflammatory cytokines, thus strengthening the CS [2-4]. These feedback loops represent the typical vicious circle (Figure 2).

Our therapeutic strategy was directed to block virus penetration by inhibiting TMPRSS2 (with BRH by inhalation), to inhibit NLRP3 inflammasome and CS, respectively (with colchicine), and to inhibit HA synthesis, respectively, in the HA storm (with hymecromone) (Figure 2). Initiation of this treatment combination as early as possible is crucial.

The present case demonstrates the clinical effectiveness of our unique therapeutic regimen, dedicated to preventing the development of a cytokine storm instead of fighting its consequences. We have shown that adding the combination therapy of inhalatory BRH, colchicine, and hymecromone to the standard therapy leads to a rapid clinical recovery and improvement of radiological images and laboratory values. With the exception of transient mild diarrhea on day 3, we did not observe significant complications during the treatment process.

A tubulin inhibitor and a microtubule disrupting agent, colchicine, theoretically has a number of anti-SARS-CoV-2 effects. Colchicine is used to treat gout and Behcet’s disease, but it has been also used as a potent anti-inflammatory medication that causes inhibition of neutrophil chemotaxis, adhesion and mobilization, inflammasome inhibition, and tumor necrosis factor (TNF) reduction and can interfere with several inflammatory pathways [2].

A number of clinical trials have been conducted, two of which were widely cited, large-scale studies - Recovery and ColCorona [7,8]. However, the results were least to say contradictory, ranging from no effect to very limited effect with conclusions that “Colchicine is not a silver bullet” for COVID-19 [7,8]. Our experience and results showed that the equivocal findings in these studies are due to the lower therapeutic doses used. Applying the so-called standard doses of 1 mg per day has practically no effect on the treatment of COVID-19 patients. However, there is no clarity or consensus in the literature on what is the standard or optimal colchicine dose, even in well-known conditions such as gout [2]. The most common and significant adverse effect of colchicine is diarrhea [2]. Our results and non-published data clearly demonstrated that an excellent therapeutic effect can be achieved by higher doses of colchicine. А pronounced effect is observed at a colchicine loading dose calculated by the following formula: 0.5 mg/per 10 kg body weight - 0.5 mg (not more than 5 mg per day). This is followed by a daily maintenance dose that is half of the loading dose. Our therapeutic regimen combining higher doses of colchicine, inhalatory BRH, and hymecromone resulted in a mortality rate as low as 3.846% (n=3 of 78 patients). Our control group of 242 patients treated only with the standard regimen, including steroids, heparin, oxygen, and antibiotics, had a mortality rate of 18.18%. Another similar therapeutic regimen (first day 3.5-4 mg colchicine, second day 3 mg, third day 2.5 mg, 4-6 days 1.5 mg) used in 322 patients showed a mortality rate of 4.34% compared to 21.126% in the control group of 71 patients [9].

High doses of colchicine similar to those used in our case have been used before with diarrhea being the only significant side effect in up to 76% [10]. However, the therapeutic benefits shown in our case overwhelmingly outweighed this non-life-threatening, easily corrected, side effect, i.e., mild diarrhea. It remains unclear why large-scale and widely promoted clinical studies, such as Recovery and ColCorona, have not tested the effect of high doses of colchicine similar to other randomized, double-blind clinical trials such as the one conducted by Terkeltaub et al. [10].

Severe COVID-19 is characterized by an aggressive inflammatory response, leading to the release of a large amount of pro-inflammatory cytokines, including IL-1β and TNF-α [9,10]. The levels of IL-1β and TNF-α, which are strong inducers of the major enzyme responsible for hyaluronic acid/hyaluronan (HA) synthesis, hyaluronan synthase 2 (HAS2), are high in the lungs of COVID-19 patients [11]. Five fully conserved “human identical sequences (HIS)”, identified in the SARS-CoV-2 genome, directly interact with host enhancers to activate the expression of cytokine genes, ACE2 and HAS2, which further increases HA formation [3]. The SARS-CoV2 infection causes accumulation of HA (induces hyaluronan storm syndrome), and thus participating in COVID-19 ARDS [3,4]. These events are very similar to severe cases of influenza [12].

HA is a nonsulfated glycosaminoglycan made up of D-glucuronic acid and N-acetyl-D-glucosamine, whose expression is elevated in a variety of tumors as in prostate cancer [13]. In normal lungs, HA is present in intact alveolar walls and perivascular tissue. Very importantly, HA has the ability to trap water up to 1000 times its molecular weight and when bound to water, the resulting hydrogel obtains a stiff viscous quality similar to ‘Jello’ [14]. The HA accumulation in the alveolar spaces of the lungs is correlated with the occurrence of hypoxemia and respiratory failure in the critical patient group. HA obstructs alveoli with a presence in exudate and plugs, as well as in thickened perialveolar interstitium [15]. In fatal COVID-19 cases, a liquid jelly of prominent hyaluronan exudates has been found in the alveolar spaces at autopsy, much resembling the lungs of wet drowning [4,14]. The HA plasma level is tightly correlated with severity and high risk for ARDS and may act as a predictor for the progression of COVID-19 [16].

The first step in the HA synthesis is the transfer of a uridine diphosphate-residue to N-acetylglucosamine and glucuronic acid via the UDP-glucuryltransferase (UGT), forming, in this way, the precursors UDP-glucuronic acid (UDP-GlcUA) and UDP-N-acetyl-glucosamine (UDP-GlcNAc). The second step is the synthesis of HA by HAS1, HAS2, and HAS3 from these precursors. Each of the HASs, which are integral membrane proteins, synthesizes HA of different molecular masses [14].

The natural product, found in many plants, coumarin, hydroxylated at position 7, is known as umbelliferone [17]. A derivative of coumarin, 4-MU (4-Methylumbelliferone; 7-hydroxy-4-methylcoumarin; hymecromone), a clinically approved bile therapy drug with choleretic and antispasmodic properties, acts on the first step of HA synthesis as a competitive inhibitor of UGT. 4MU also downregulates HAS2 and HAS3 expression and thus affects the second step in HA synthesis [13]. 4-MU also reduces the inflammatory cytokine level. In addition, lipopolysaccharide-induced lung inflammation can be relieved by 4-MU. Therefore, HA could be a novel target for COVID-19 treatment, and inhibiting the production of HA could help COVID-19 patients breathe. Our unpublished data analysis of combination therapy with inhallatory BRH, colchicine, and hymecromone showed that immediate initiation of the therapy in outpatient settings virtually eliminates the risk of complications and hospital admission [18,19].

## Conclusions

Our experience of more than two years with the combination therapeutic regimen, including inhallatory BRH, higher doses of colchicine, and hymecromone, clearly indicates that immediate administration of the therapy practically eliminates the risk of complications in outpatient settings and decreases mortality rates in inpatient settings four to five-fold. We strongly recommend that the treatment of outpatients with COVID-19 begins immediately with bromhexine or ambroxol inhalation. This can be done even in contact with infected people and will greatly limit the penetration of the virus into the cells. In parallel, taking colchicine at the doses we recommend will prevent the eventual development of a cytokine storm.
